# *N*,*N*-Diethyl-*N*′-phenyl­acetyl­thio­urea

**DOI:** 10.1107/S1600536808035290

**Published:** 2008-11-08

**Authors:** Liang Xian

**Affiliations:** a Chemical Engineering Institute, Northwest University for Nationalities, Lanzhou 730124, People’s Republic of China

## Abstract

The title thio­urea mol­ecule, C_13_H_18_N_2_OS, adopts a folded conformation due to the steric hindrance of the two ethyl groups and the acetyl group. In the crystal structure, the acetyl O atom is not involved in hydrogen bonding, but inter­molecular N—H⋯S hydrogen bonds link the mol­ecules into centrosymmetric dimers.

## Related literature

For general background on the chemistry of thio­urea derivatives, see: Choi *et al.* (2008 ▶); Jones *et al.* (2008 ▶); Kushwaha *et al.* (2008 ▶); Su *et al.* (2006 ▶). For related structures, see: Su (2005 ▶, 2007 ▶); Xian *et al.* (2004 ▶); Xian (2008 ▶).
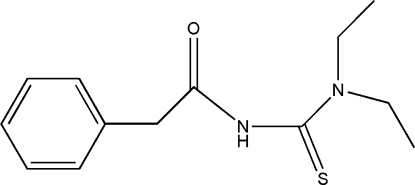


## Experimental

### 

#### Crystal data

C_13_H_18_N_2_OS
*M* *_r_* = 250.35
Monoclinic, 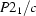

*a* = 11.578 (7) Å
*b* = 12.804 (8) Å
*c* = 9.176 (6) Å
β = 103.842 (10)°
*V* = 1320.8 (15) Å^3^
*Z* = 4
Mo *K*α radiation
μ = 0.23 mm^−1^
*T* = 296 (2) K
0.30 × 0.29 × 0.25 mm

#### Data collection

Bruker SMART CCD area-detector diffractometer
Absorption correction: multi-scan (*SADABS*; Sheldrick, 2000 ▶) *T*_min_ = 0.933, *T*_max_ = 0.944
7619 measured reflections
3080 independent reflections
2484 reflections with *I* > 2σ(*I*)
*R*_int_ = 0.028

#### Refinement

*R*[*F* ^2^ > 2σ(*F* ^2^)] = 0.042
*wR*(*F* ^2^) = 0.124
*S* = 1.05
3080 reflections
156 parameters
H-atom parameters constrained
Δρ_max_ = 0.48 e Å^−3^
Δρ_min_ = −0.37 e Å^−3^

### 

Data collection: *APEX2* (Bruker, 2001 ▶); cell refinement: *APEX2* and *SAINT* (Bruker, 2001 ▶); data reduction: *SAINT*; program(s) used to solve structure: *SHELXS97* (Sheldrick, 2008 ▶); program(s) used to refine structure: *SHELXL97* (Sheldrick, 2008 ▶); molecular graphics: *SHELXTL* (Sheldrick, 2008 ▶); software used to prepare material for publication: *SHELXTL*.

## Supplementary Material

Crystal structure: contains datablocks global, I. DOI: 10.1107/S1600536808035290/cv2467sup1.cif

Structure factors: contains datablocks I. DOI: 10.1107/S1600536808035290/cv2467Isup2.hkl

Additional supplementary materials:  crystallographic information; 3D view; checkCIF report

## Figures and Tables

**Table 1:** Hydrogen-bond geometry (Å, °)

*D*—H⋯*A*	*D*—H	H⋯*A*	*D*⋯*A*	*D*—H⋯*A*
N1—H1′⋯S1^i^	0.86	2.69	3.404 (3)	141

Symmetry code: (i) 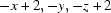.

**Fig. 1.**
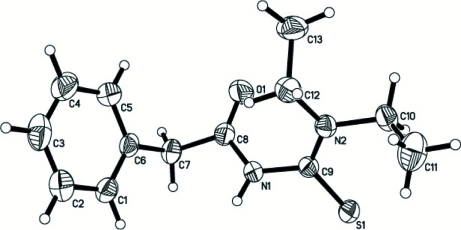
The molecular structure of the title compound. Displacement ellipsoids are drawn at the 40% probability level.

**Fig. 2.**
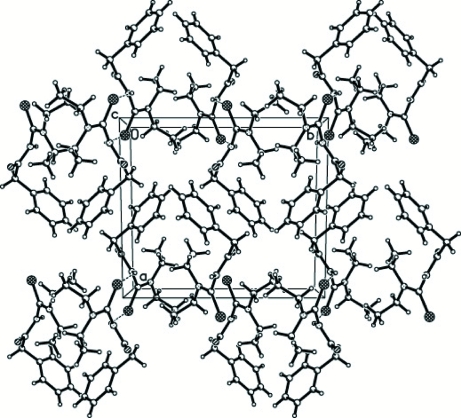
Packing diagram of the title compound viewed along the c axis. Intermolecular hydrogen bonds are shown as dashed lines.

## supplementary crystallographic information

### Comment

Thiourea and its derivatives attract special attention in recent years because of their broad applications, such as anion recognition, nonlinear optical material, catalysis *etc*., and also due to high bioactivity and good coordination ability (Choi *et al.*, 2008; Kushwaha *et al.*, 2008; Jones *et al.* 2008; Su *et al.*, 2006). For a long time, we have being interested in the influence of non-covalent interactions related to the substituted groups on the conformations of thiourea derivatives as well as their coordination abilities. Thiourea derivatives with different substituted groups coordinate different transition metal ions providing various structures. One of the key influence factors in coordination reactions is non-covalent interaction. However, the central ion also plays and important role. Triangle conformation is commonly observed in the coordination compound of benzoylthiourea with Cu(I) (Xian *et al.*, 2004). However, Cu_6_ cluster structure was also obtained (Su *et al.*, 2005). Herewith we present the crystal structure of the title compound, (I).

The conformation and the packing diagram of (I) are shown in Figures 1 and 2, respectively. It can be seen that the title compound has a folded conformation which is similar to the structure we obtained before (CCDC No. 699688). The dihedral angle between the benzene ring and the plane O1/ N1/C7/C8 is 69.12 (6)°, and the dihedral angle between the benzene ring and the plane S1/C9/N1/N2 is 67.19 (6)°. Apparently, stereo-hindrance effect of two ethyl groups and acetyl group is the main influence factor to the folded conformation. Because of the absence of hydrogen atom on N2, the acetyl oxygen atom does not take part in hydrogen bonding interactions. This is different from the other carbonylthiourea derivatives (Su *et al.*, 2006; Xian, 2008), in which the carbonyl oxygen atom often forms a six-membered hydrogen bonding ring. However, thiocarbonyl sulfur atom is involved in an intermolecular N—H···S hydrogen bond (Table 1), linking two molecules into centrosymmetric dimer, that was eralier observed in related structures (Su, 2007; Xian, 2008).

### Experimental

All reagents and organic solvents were of analytical reagent grade and commercially available. Phenylacetyl chloride (1.55 g) was treated with ammonium thiocyanate (1.20 g) in CH_2_Cl_2_ under solid-liquid phase transfer catalysis conditions, using 3% polyethylene glycol-400 as catalyst, to give the corresponding phenylacetyl isothiocyanate, which was reacted with diethylamine (0.73 g) to give the title compound. The solid was separated from the liquid phase by filtration, washed with CH_2_Cl_2_ and then dried in air. Colorless single crystals suitable for X-ray analysis were obtained after one week by slow evaporation of an chloroform solution. The infrared spectrum was recorded in the range of 4000–400 cm^-1^ on a Nicolet NEXUS 670 F T—IR spectrometer, using KBr pellets. ^1^H NMR spectrum was obtained on an INOVA-400 MHz superconduction spectrometer, acetone_d6_ was used as solvent and TMS as internal standard, and the chemical shifts are expressed as delta. Elemental analyses were carried out on a PE-2400 elemental analysis instrument. Melting point determination was performed in YRT-3 melting point instrument (Tianjin) and was uncorrected. Melting Point: 92–94 °C. Elemental analysis (%) found (calcd.): C, 62.3(60.5); H, 7.2(6.9); N, 11.2(13.6); S, 12.8(10.9). IR (KBr, cm^-1^): 3190 (N—H), 3079, 1711 (C=O), 1548(C=C), 1233(C=S), 1121. ^1^H NMR(delta, p.p.m.): 2.06 (m, 3H, CH_3_); 2.85 (m, 3H, CH_3_);3.70 (m, 6H, 3CH_2_); 7.22–7.38 (m, 5H, C_6_H_5_); 9.25 (s, 1H, NH).

### Refinement

All H atoms were placed in calculated positions (C–H = 0.93–0.97 Å, N–H = 0.86 Å) and refined using the riding model approximation, with *U*_iso_(H) = 1.2 or 1.5 *U*_eq_ of the parent atom.

### Crystal data

**Table:**

C_13_H_18_N_2_OS	*F*_000_ = 536
*M**_r_* = 250.35	*D*_x_ = 1.259 Mg m^−^^3^
Monoclinic, *P*2_1_/*c*	Mo *K*α radiation λ = 0.71073 Å
*a* = 11.578 (7) Å	Cell parameters from 3844 reflections
*b* = 12.804 (8) Å	θ = 2.4–29.9º
*c* = 9.176 (6) Å	µ = 0.23 mm^−^^1^
β = 103.842 (10)º	*T* = 296 (2) K
*V* = 1320.8 (15) Å^3^	Block, colorless
*Z* = 4	0.30 × 0.29 × 0.25 mm

### Data collection

**Table:**

Bruker SMART CCD area-detector diffractometer	3080 independent reflections
Radiation source: fine-focus sealed tube	2484 reflections with *I* > 2σ(*I*)
Monochromator: graphite	*R*_int_ = 0.028
*T* = 296(2) K	θ_max_ = 28.0º
φ and ω scans	θ_min_ = 1.8º
Absorption correction: multi-scan(SADABS; Sheldrick, 2000)	*h* = −15→13
*T*_min_ = 0.934, *T*_max_ = 0.944	*k* = −16→16
7619 measured reflections	*l* = −12→11

### Refinement

**Table:**

Refinement on *F*^2^	Secondary atom site location: difference Fourier map
Least-squares matrix: full	Hydrogen site location: inferred from neighbouring sites
*R*[*F*^2^ > 2σ(*F*^2^)] = 0.042	H-atom parameters constrained
*wR*(*F*^2^) = 0.124	*w* = 1/[σ^2^(*F*_o_^2^) + (0.0624*P*)^2^ + 0.2848*P*] where *P* = (*F*_o_^2^ + 2*F*_c_^2^)/3
*S* = 1.05	(Δ/σ)_max_ < 0.001
3080 reflections	Δρ_max_ = 0.48 e Å^−^^3^
156 parameters	Δρ_min_ = −0.37 e Å^−^^3^
Primary atom site location: structure-invariant direct methods	Extinction correction: none

### Special details

**Table:**

Geometry. All e.s.d.'s (except the e.s.d. in the dihedral angle between two l.s. planes) are estimated using the full covariance matrix. The cell e.s.d.'s are taken into account individually in the estimation of e.s.d.'s in distances, angles and torsion angles; correlations between e.s.d.'s in cell parameters are only used when they are defined by crystal symmetry. An approximate (isotropic) treatment of cell e.s.d.'s is used for estimating e.s.d.'s involving l.s. planes.
Refinement. Refinement of *F*^2^ against ALL reflections. The weighted *R*-factor *wR* and goodness of fit *S* are based on *F*^2^, conventional *R*-factors *R* are based on *F*, with *F* set to zero for negative *F*^2^. The threshold expression of *F*^2^ > σ(*F*^2^) is used only for calculating *R*-factors(gt) *etc*. and is not relevant to the choice of reflections for refinement. *R*-factors based on *F*^2^ are statistically about twice as large as those based on *F*, and *R*- factors based on ALL data will be even larger.

### Fractional atomic coordinates and isotropic or equivalent isotropic displacement parameters (Å^2^)

**Table:**

	*x*	*y*	*z*	*U*_iso_*/*U*_eq_	
C1	0.61035 (14)	0.09311 (14)	0.9820 (2)	0.0472 (4)	
H1	0.6752	0.0776	1.0607	0.057*	
C2	0.53176 (18)	0.17042 (15)	0.9993 (2)	0.0570 (5)	
H2	0.5441	0.2071	1.0892	0.068*	
C3	0.43555 (19)	0.19328 (17)	0.8842 (3)	0.0663 (6)	
H3	0.3821	0.2449	0.8962	0.080*	
C4	0.41832 (18)	0.1398 (2)	0.7515 (3)	0.0716 (6)	
H4	0.3531	0.1552	0.6733	0.086*	
C5	0.49718 (16)	0.06344 (17)	0.7335 (2)	0.0577 (5)	
H5	0.4856	0.0280	0.6426	0.069*	
C6	0.59380 (13)	0.03890 (13)	0.84970 (19)	0.0406 (4)	
C7	0.67692 (14)	−0.04934 (13)	0.8329 (2)	0.0457 (4)	
H7A	0.7249	−0.0684	0.9311	0.055*	
H7B	0.6303	−0.1099	0.7909	0.055*	
C8	0.75749 (14)	−0.01928 (13)	0.73300 (19)	0.0426 (4)	
C9	0.95069 (13)	0.06202 (11)	0.73941 (17)	0.0359 (3)	
C10	1.02328 (16)	0.14815 (14)	0.5447 (2)	0.0488 (4)	
H10A	0.9893	0.1590	0.4384	0.059*	
H10B	1.0782	0.0899	0.5550	0.059*	
C11	1.0909 (2)	0.24531 (17)	0.6105 (3)	0.0755 (6)	
H11A	1.0370	0.3033	0.5999	0.113*	
H11B	1.1517	0.2601	0.5583	0.113*	
H11C	1.1268	0.2340	0.7149	0.113*	
C12	0.81587 (16)	0.18024 (14)	0.5634 (2)	0.0491 (4)	
H12A	0.8331	0.2545	0.5695	0.059*	
H12B	0.7629	0.1657	0.6284	0.059*	
C13	0.75285 (19)	0.15330 (19)	0.4033 (2)	0.0675 (6)	
H13A	0.7990	0.1781	0.3363	0.101*	
H13B	0.6759	0.1858	0.3788	0.101*	
H13C	0.7438	0.0789	0.3935	0.101*	
N1	0.85660 (11)	0.03650 (10)	0.80451 (15)	0.0398 (3)	
H1'	0.8610	0.0570	0.8949	0.048*	
N2	0.92772 (11)	0.12212 (10)	0.61873 (14)	0.0387 (3)	
O1	0.73627 (12)	−0.04082 (12)	0.60141 (15)	0.0640 (4)	
S1	1.08598 (3)	0.01581 (4)	0.82130 (5)	0.04902 (16)	

### Atomic displacement parameters (Å^2^)

**Table:**

	*U*^11^	*U*^22^	*U*^33^	*U*^12^	*U*^13^	*U*^23^
C1	0.0353 (8)	0.0537 (9)	0.0528 (10)	−0.0047 (7)	0.0107 (7)	0.0001 (8)
C2	0.0569 (11)	0.0526 (10)	0.0681 (12)	−0.0026 (8)	0.0276 (9)	−0.0033 (9)
C3	0.0603 (12)	0.0608 (11)	0.0887 (16)	0.0197 (10)	0.0394 (11)	0.0248 (11)
C4	0.0507 (11)	0.0962 (16)	0.0667 (13)	0.0215 (11)	0.0120 (10)	0.0285 (12)
C5	0.0466 (10)	0.0793 (13)	0.0460 (10)	0.0026 (9)	0.0088 (8)	0.0047 (9)
C6	0.0312 (7)	0.0456 (8)	0.0473 (9)	−0.0053 (6)	0.0135 (6)	0.0047 (7)
C7	0.0391 (8)	0.0452 (8)	0.0553 (10)	−0.0041 (7)	0.0166 (7)	0.0006 (7)
C8	0.0372 (8)	0.0469 (8)	0.0445 (9)	0.0000 (7)	0.0114 (7)	−0.0029 (7)
C9	0.0347 (7)	0.0387 (7)	0.0351 (7)	−0.0001 (6)	0.0101 (6)	−0.0047 (6)
C10	0.0481 (9)	0.0569 (10)	0.0460 (9)	−0.0014 (8)	0.0203 (7)	0.0064 (8)
C11	0.0678 (13)	0.0557 (11)	0.1072 (19)	−0.0136 (10)	0.0293 (13)	0.0072 (12)
C12	0.0502 (9)	0.0500 (9)	0.0473 (9)	0.0154 (7)	0.0124 (7)	0.0034 (7)
C13	0.0625 (12)	0.0880 (15)	0.0467 (10)	0.0285 (11)	0.0026 (9)	0.0024 (10)
N1	0.0344 (6)	0.0527 (8)	0.0340 (6)	−0.0010 (5)	0.0115 (5)	−0.0037 (6)
N2	0.0375 (7)	0.0411 (7)	0.0386 (7)	0.0032 (5)	0.0115 (5)	−0.0004 (5)
O1	0.0590 (8)	0.0866 (10)	0.0472 (8)	−0.0209 (7)	0.0143 (6)	−0.0180 (7)
S1	0.0346 (2)	0.0687 (3)	0.0442 (3)	0.00839 (18)	0.01022 (17)	0.00658 (19)

### Geometric parameters (Å, °)

**Table:**

C1—C6	1.371 (2)	C9—N1	1.401 (2)
C1—C2	1.379 (3)	C9—S1	1.6747 (17)
C1—H1	0.9300	C10—N2	1.469 (2)
C2—C3	1.371 (3)	C10—C11	1.516 (3)
C2—H2	0.9300	C10—H10A	0.9700
C3—C4	1.370 (4)	C10—H10B	0.9700
C3—H3	0.9300	C11—H11A	0.9600
C4—C5	1.374 (3)	C11—H11B	0.9600
C4—H4	0.9300	C11—H11C	0.9600
C5—C6	1.384 (2)	C12—N2	1.474 (2)
C5—H5	0.9300	C12—C13	1.515 (3)
C6—C7	1.516 (2)	C12—H12A	0.9700
C7—C8	1.506 (2)	C12—H12B	0.9700
C7—H7A	0.9700	C13—H13A	0.9600
C7—H7B	0.9700	C13—H13B	0.9600
C8—O1	1.205 (2)	C13—H13C	0.9600
C8—N1	1.377 (2)	N1—H1'	0.8600
C9—N2	1.322 (2)		
			
C6—C1—C2	120.61 (17)	N2—C10—C11	112.09 (16)
C6—C1—H1	119.7	N2—C10—H10A	109.2
C2—C1—H1	119.7	C11—C10—H10A	109.2
C3—C2—C1	120.1 (2)	N2—C10—H10B	109.2
C3—C2—H2	120.0	C11—C10—H10B	109.2
C1—C2—H2	120.0	H10A—C10—H10B	107.9
C4—C3—C2	119.81 (19)	C10—C11—H11A	109.5
C4—C3—H3	120.1	C10—C11—H11B	109.5
C2—C3—H3	120.1	H11A—C11—H11B	109.5
C3—C4—C5	120.2 (2)	C10—C11—H11C	109.5
C3—C4—H4	119.9	H11A—C11—H11C	109.5
C5—C4—H4	119.9	H11B—C11—H11C	109.5
C4—C5—C6	120.4 (2)	N2—C12—C13	113.49 (14)
C4—C5—H5	119.8	N2—C12—H12A	108.9
C6—C5—H5	119.8	C13—C12—H12A	108.9
C1—C6—C5	118.89 (17)	N2—C12—H12B	108.9
C1—C6—C7	120.65 (15)	C13—C12—H12B	108.9
C5—C6—C7	120.42 (17)	H12A—C12—H12B	107.7
C8—C7—C6	111.86 (14)	C12—C13—H13A	109.5
C8—C7—H7A	109.2	C12—C13—H13B	109.5
C6—C7—H7A	109.2	H13A—C13—H13B	109.5
C8—C7—H7B	109.2	C12—C13—H13C	109.5
C6—C7—H7B	109.2	H13A—C13—H13C	109.5
H7A—C7—H7B	107.9	H13B—C13—H13C	109.5
O1—C8—N1	122.88 (15)	C8—N1—C9	124.21 (14)
O1—C8—C7	122.95 (16)	C8—N1—H1'	117.9
N1—C8—C7	114.16 (15)	C9—N1—H1'	117.9
N2—C9—N1	118.15 (13)	C9—N2—C10	119.78 (13)
N2—C9—S1	124.09 (12)	C9—N2—C12	124.49 (13)
N1—C9—S1	117.75 (12)	C10—N2—C12	115.02 (14)
			
C6—C1—C2—C3	0.4 (3)	O1—C8—N1—C9	−8.9 (3)
C1—C2—C3—C4	−0.6 (3)	C7—C8—N1—C9	172.09 (14)
C2—C3—C4—C5	0.0 (3)	N2—C9—N1—C8	62.2 (2)
C3—C4—C5—C6	0.8 (3)	S1—C9—N1—C8	−118.74 (15)
C2—C1—C6—C5	0.4 (2)	N1—C9—N2—C10	−178.25 (14)
C2—C1—C6—C7	−177.23 (15)	S1—C9—N2—C10	2.7 (2)
C4—C5—C6—C1	−1.0 (3)	N1—C9—N2—C12	11.9 (2)
C4—C5—C6—C7	176.60 (17)	S1—C9—N2—C12	−167.16 (12)
C1—C6—C7—C8	−107.78 (18)	C11—C10—N2—C9	−88.3 (2)
C5—C6—C7—C8	74.7 (2)	C11—C10—N2—C12	82.5 (2)
C6—C7—C8—O1	−96.5 (2)	C13—C12—N2—C9	−122.62 (19)
C6—C7—C8—N1	82.50 (18)	C13—C12—N2—C10	67.1 (2)

### Hydrogen-bond geometry (Å, °)

**Table:**

*D*—H···*A*	*D*—H	H···*A*	*D*···*A*	*D*—H···*A*
N1—H1'···S1^i^	0.86	2.69	3.404 (3)	141

Symmetry codes: (i) −*x*+2, −*y*, −*z*+2.
